# Diabetes knowledge predicts HbA1c levels of people with type 2 diabetes mellitus in rural China: a ten-month follow-up study

**DOI:** 10.1038/s41598-023-45312-y

**Published:** 2023-10-25

**Authors:** Xiaoying Wang, Bo Tian, Shengfa Zhang, Jinsui Zhang, Weiping Yang, Jina Li, Weiwei Wang, Yuchen Wang, Weijun Zhang

**Affiliations:** 1 School of Social Development and Public Policy, Center for Behavioral Health, Beijing Normal University, Beijing, China; 2 National Population Heath Data Center, Chinese Academy of Medical Sciences and Peking Union Medical College, Beijing, China; 3 School of Public Health, Fudan University, Shanghai, China; 4 Yancheng Dafeng People’s Hospital, Yancheng, Jiangsu Province China; 5 School of Sociology and Population Studies, Renmin University of China, Beijing, China; 6 North China Electric Power University, Beijing, China

**Keywords:** Patient education, Type 2 diabetes

## Abstract

Improving diabetes self-management (DSM) is facing real-world challenges among people with type 2 diabetes mellitus (T2DM) who have a low education level in resource-limited areas. This study aimed to investigate whether diabetes knowledge could predict glycemic levels in people with T2DM in rural China. This analytical cross-sectional study recruited 321 people with T2DM from eight villages by purposive sampling at baseline. After 10 months, 206 patients completed the follow-up survey and HbA1c tests, with a response rate of 64.17% (206/321). Multiple regression analysis was employed to explore the correlation between diabetes knowledge and HbA1c levels. The patient's diabetes knowledge was significantly negatively correlated with HbA1c levels before and after controlling for covariates in both hierarchical multiple regression and multiple logistic regression (*p* < 0.01). In addition, other influencing factors, including sex, age, marital status, employment status, income, and HbA1c levels at baseline, were also identified. Diabetes knowledge could predict HbA1c levels significantly among patients with low education levels in rural China. Therefore, interventions on improving diabetes knowledge need to be strengthened for patients in rural China so that they can improve their health outcomes and reduce the disease burden.

## Introduction

The prevalence of type 2 diabetes mellitus (T2DM) has sharply increased in the past four decades^1^. Diabetes and its complications not only seriously affect patients’ health but also bring huge economic burdens to patients, their families, and society. In 2019, China’s medical expenditures related to diabetes reached $109 billion, ranking second in the world^2^. To prevent and control diabetes, numerous studies were conducted and confirmed that lifestyle factors could influence patients’ health outcomes^3–5^. Furthermore, several studies, which were conducted in China, Finland, and America, found that lifestyle interventions, including diet and exercise, can postpone the onset of T2DM, reduce the incidence of diabetes complications, and ultimately increase the life expectancy of people with T2DM^6–11^. To apply existing and extensive evidence to manage diabetes in primary care, the International Diabetes Federation (IDF) emphasized that the cornerstone of T2DM management is to improve diabetes self-management (DSM) ability, including diet, medication adherence, physical activity, and healthy body weight and has produced a series of guidelines on diabetes management, prevention, and care^12,13^.

China has the largest number of people with diabetes^1^ and thus may face the considerable challenge of chronic complications in the future. The government has already paid much attention to managing diabetes and enacted the *policy of equalization of basic public health services* in 2009^14^. In rural China, people with T2DM are targeted as the key population for chronic disease management and the specific measures include regular quarterly follow-ups, free fasting blood glucose (FBG) tests, an annual comprehensive health examination, health education, and different medical prescriptions for people with T2DM^15^. Although the policy has been implemented for almost 12 years, the challenge related to diabetes prevention and control remains large and serious^16,17^. A recent study estimated that only 49.2% of treated patients achieved successful glycemic control in China (HbA1c levels ≤ 7.0%)^18^. Another study showed that patients’ DSM behavior scores were at a lower-middle level in a suburban hospital in Beijing^19^. Similarly, a study conducted in Shandong Province found that, compared with patients from urban areas, patients from rural areas had poorer DSM behaviors^20^. Patients in rural China are facing greater difficulties and challenges than those in urban areas because of their lower education levels^21^ and an unbalanced distribution of high-quality medical resources^22^. Therefore, improving the DSM of people with T2DM through educational interventions in rural China should be a top priority now and in the future^23^.

HbA1c, which reflects average plasma glucose over 2 to 3 months preceding the test, has been not only considered as a biomarker for the presence and severity of hyperglycemia, implying diabetes or pre-diabetes^24^, but also considered as a risk factor marker for diabetes-related complications^25^ in the process of diabetes treatment and management. Serval studies indicated that high HbA1c variability is not only associated with cardiovascular complications of T2DM^26,27^ but also associated with increased risk of all-cause and cardiovascular mortality^25^. Therefore, HbA1c was used as a biochemical marker of glucose regulation in people with T2DM^28^, and it was also an outcome variable in the diabetes prevention intervention program^29^. Similarly, HbA1c will also be an important outcome variable in our future intervention studies, aiming to reflect glycemic management in patients with diabetes.

Before the intervention, the relationship between diabetes knowledge and HbA1c levels should be cleared. Although there have been a few studies investigating the effects of the policy^30,31^, which indicated changes in diabetes knowledge, medication compliance, DSM, and HbA1c levels, little attention has been given to further exploring the association between diabetes knowledge and HbA1c levels in patients in rural China. Besides, these studies have used cross-sectional data^32–34^, which cannot do the causal inference.

Therefore, this analytical cross-sectional study aimed to investigate whether diabetes knowledge could predict the HbA1c levels of people with T2DM who have a low education level in rural China based on tracking data.

## Materials and methods

### Participants

The baseline survey was conducted from January 4 to January 17, 2020, in eight villages of three towns in DaFeng District, Jiangsu Province. Participants who were diagnosed with T2DM based on their electronic health records in each village clinic were involved in this study. Inclusion criteria: (1) a diagnosis of T2DM from a hospital at a secondary level and above, based on *Guidelines for the Prevention and Treatment of Type 2 Diabetes in China*^35^; (2) 18 years old and above; and (3) continuous residence for more than one year. Exclusion criteria included the inability to participate due to physical/mental disabilities or cognitive impairment. Eventually, a sample of 321 participants was recruited into the study.

### Sampling

In China, there is a five-tier administrative system, including provinces, cities, counties/districts, towns, and villages/communities^36^. Purposive sampling was employed in this study: (1) Dafeng District was chosen as the site because it is highly representative from the perspective of economic development level and is a National Demonstration Area for comprehensive prevention and control of chronic diseases^37^. (2) Three towns, including W town, X town, and D town, were selected based on the results of the performance assessment conducted by the local health bureau. (3) Two villages from W town, four villages from X town, and one village from D town were selected based on the population size of the town. (4) All people with T2DM who registered in the village clinic and met the inclusion criteria were interviewed face-to-face by trained interviewers. Since there were no related studies on the effect size between diabetes knowledge and HbA1c, we chose a small effect size (0.15)^38^ to achieve a maximum sample size. A priori G*power 3.1^39^ calculations revealed a minimum sample size of 117 participants within a multiple regression analysis with 5 predictors to detect a small effect size, using α = 0.05, power (1-β) = 0.90, and effect size = 0.15. Taking into account a 20% loss-to-follow-up rate, the final total sample size was 141 participants.

### Procedure for field survey

The patients who volunteered to participate in the study were invited to village clinics, and four well-educated graduate students conducted the self-report questionnaire. The village doctors at village clinics were responsible for the HbA1c test. Considering that most participants, with low levels of education, cannot speak Mandarin, four trained volunteers from a local voluntary organization were invited to solve dialect barriers.

Three hundred and twenty-one participants completed the baseline survey. Ten months later, 206 participants completed the follow-up investigation and received HbA1c tests, and the attrition rate was 35.83% (115/321). There were no significant differences between the follow-up samples and loss to follow-up samples sample in terms of age (t = −0.504, *p* = 0.630), HbA1c level at baseline (t = 0.520, *p* = 0.603), and diabetes knowledge (t = −0.512, *p* = 0.609), except that the higher proportion of females was found in respondents (χ^2^ = 10.137, *p* = 0.001). Therefore, the loss to follow-up did not affect the stability of the relationship between diabetes knowledge and HbA1c levels.

### Measurements

#### Demographic information and clinical characteristics

The information, including age, sex, education level, marital status, employment status, and annual household income, was collected during the baseline survey. Clinical characteristics, including a family history of diabetes, body mass index (BMI), duration of diabetes, hypoglycemia, and diabetes complications, including hypertension, cardiac disease, diabetic nephropathy, diabetic retinopathy, diabetic peripheral angiopathy, and others, were obtained from the electronic health record system at township health centers.

#### Diabetes knowledge

Diabetes knowledge was assessed by the modified 20-item Diabetes Knowledge scale at baseline, which combined the original version of Diabetes Knowledge scale (DKN)^40^ with the Chinese version of DKN scale^41^. Taking into account the literacy level and lifestyle of the participants, we fine-tuned the scale. The process can be found in the Appendix. The final version of the DKN scale consisted of 17 single-choice items and 3 multiple-choice items. Each item is assigned a score of one for a correct answer and 0 for an incorrect or unknown response. A higher score suggested a higher level of diabetes knowledge. Cronbach’s alpha of the modified 20-item DKN scale in this sample was 0.73.

#### HbA1c test

HbA1c level reflects the average blood glucose concentrations for the preceding 2–3 months in patients^42,43^. In this study, HbA1c levels were tested, at baseline (T1) and 10-month (T2) follow-up surveys, by using the portable HbA1c meter and the Diagnosis Kit for Human Glycosylated Hemoglobin (Botangping in Chinese).

#### Medication adherence

Medication adherence was measured at T2 using a self-designed questionnaire based on a thesis about medication adherence in Chinese people with heart failure^44^, which includes eight items concerning the situation of forgetting to take medicine (four items), unauthorized withdrawal of taking medicine (two items), and perceived difficulty in taking prescribed medication (one item). Each item was designed with a five-point scale ranging from 1 to 5, and the total score was 40. If a participant’s score was equal to 40, it was defined as “medication adherence”; otherwise, it was defined as “medication nonadherence”. In this study, Cronbach’s alpha of the self-designed questionnaire was 0.93.

#### Diabetes self-management

An adapted version of the Diabetes Self-care Activities (SDSCA) measure, which consists of items covering diet, exercise, blood sugar testing, foot care, and smoking, was used to assess the level of DSM in this study^45^. After the pilot study, some changes were made as follows: (1) The term “checking your foot” was removed because participants could not understand and communicate the true meaning. (2) Several specific food names were supplemented behind the word “high-fat food” to ensure participants’ better understanding of the item. Finally, the adapted version of the SDSCA measure included 7 items from three dimensions, and all items were measured on an eight-point scale ranging from 0 to 7. Of these, five items were about diet, one item was about exercise, and one item was about self-monitoring blood glucose (SMBG). If a participant exercised three days or more a week, he or she was deemed to have a high level of DSM; otherwise, he or she had a low level of DSM. Similarly, examining FBG twice a week or more was defined as a high level of SMBG; otherwise, it was a low level.

### Statistical analysis

To test the selection bias of the sample, Student's t-tests and Pearson’s Chi-squared tests were performed to compare the differences in sociodemographic characteristics and clinical factors between the follow-up samples and the loss to follow-up samples. Multiple regression analysis was conducted to explore the correlation between diabetes knowledge and HbA1c levels (T2) after adjusting for sociodemographic characteristics, clinical factors, and DSM. Then multiple logistic regression analysis was employed to test the stability of the relationship between diabetes knowledge and HbA1c levels.

Concurrently, regression diagnosis was conducted to examine the robustness of models. First, the residual of the two models was predicted to draw a scatter plot. The relationship between diabetes knowledge and HbA1c levels was linear. Second, eight kinds of indices were calculated to identify singular values. Seventeen singular values were found and were excluded. Third, the dependent variable satisfied a normal distribution after removing singular values. Additionally, the variance inflation factors (VIFs) of all independent variables were less than 10, which indicated that collinearity did not exist. Furthermore, the final model passed the White test, which confirmed that there was no heteroscedasticity. Finally, by calculating the cluster robust standard error, the final model satisfied the assumption of no autocorrelation.

Two individuals had missing values on BMI, which we calculated by their weight and height provided in electronic health records. Therefore, 206 participants with complete data were included in the final analyses. All analyses were conducted in Stata 14.0, and a p-value of < 0.05 was considered statistically significant.

### Ethics approval and consent to participate

Ethical approval for this study was obtained from the China Ethics Committee of Registering Clinical Trials (ChiECRCT-20180073) on June 8, 2018. All patients provided informed consent prior to the questionnaire and interview, all personal information was kept confidential, and reporting was made anonymously. All methods were carried out in accordance with relevant guidelines and regulations.

## Results

### Participant characteristics

**Figure 1:**
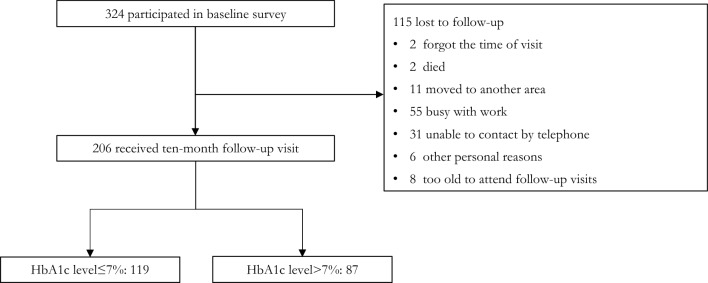
Flow diagram of numbers of individuals at each stage of study.

As shown in Fig. 1, among the participants who completed the follow-up visits, the average follow-up time was 319.25 days (SD = 10.90), which was around 10 months. The reasons why 115 participants were loss to follow-up can also be seen in Fig. 1. The sociodemographic characteristics of the respondents are shown in Table 1. Of 206 respondents, 88.83% were married and 56.8% were farmers. Most of them had a low level of education, with 25.24% being illiterate, and 36.97% only completing primary school. A majority (83.98%) had an annual household income of no more than 50,000 yuan.

**Table 1:** Sample characteristics.

	Follow-up samples (n = 206)	Loss to follow-up samples (n = 115)	*p*^*a*^
Age	64.66 ± 8.41	64.13 ± 9.84	0.614
Sex			
Male	70 (33.98)	60 (52.17)	0.001
Female	136 (66.02)	55 (47.83)	
Education level			
Uneducated	52 (25.24)	27 (23.48)	0.926
Primary school	70 (33.98)	39 (33.91)	
Junior high school or above	84 (40.78)	49 (42.61)	
Marital status			
Single	23 (11.17)	14 (12.50)	0.786
Married	183 (88.83)	101 (87.83)	
Employment status			
Farming	117 (56.80)	59 (51.30)	0.343
Other status	89 (43.20)	56 (48.70)	
Annual household income (yuan)^b^			
≤ 5,000	34 (16.50)	17 (14.78)	0.991
5,000–10,000	39 (18.93)	23 (20.00)	
10,000–20,000	51 (24.76)	28 (24.35)	
20,000–50,000	49 (23.79)	27 (23.48)	
> 50,000	33 (16.02)	20 (17.39)	

Data are presented as mean ± SD or number (percent). ^a^ Based on a Student's t-test or Pearson’s Chi-squared test.

^b^The exchange rate of Chinese Yuan in US Dollars was 0.15715 USD for 1 CNY.

### Clinical characteristics

As shown in Table 2, the average duration of diabetes was 8.11 years (SD = 5.53), with a range of 1–32 years among 206 respondents. Furthermore, 73.3% suffered from diabetes complications, and the top three complications were cardiovascular disease (72.82%), cerebrovascular disease (0.09%), and diabetic peripheral neuropathy (0.08%). No significant differences were found between the follow-up samples and loss follow-up samples sample in terms of family history of diabetes, hypoglycemia, the number of complications, duration, BMI, HbA1c levels at baseline, or diabetes knowledge.

**Table 2:** The comparison of the clinical factors between the follow-up samples and loss to follow-up samples.

	Follow-up samples (n = 206)	Loss to follow-up samples (n = 115)	*p*^*a*^
Family history of diabetes			
Yes	48 (23.30)	36 (31.30)	0.118
No	158 (76.70)	79 (68.70)	
Hypoglycemia over the past 6 months			
Yes	78 (37.86)	36 (31.30)	0.239
No	128 (62.14)	79 (68.70)	
Number of complications	0.91 ± 0.68	0.90 ± 0.78	0.839
Duration	8.11 ± 5.53	7.35 ± 5.20	0.233
BMI	26.65 ± 3.57	26.79 ± 3.41	0.726
Diabetes knowledge at baseline	10.07 ± 3.42	9.85 ± 4.16	0.608
HbA1c at baseline	7.71 ± 1.35	7.80 ± 1.70	0.603

Data are presented as mean ± SD or number (percent). ^a^ Based on a Student's t-test or Pearson’s Chi-squared test.

### Relationships between HbA1c levels and clinical outcomes

As shown in Table 3, in terms of diabetes knowledge at baseline, although the mean score of the respondents with HbA1c levels < 7% (Mean = 10.25, SD = 3.36) was higher than that of the respondents with HbA1c levels ≥ 7% (Mean = 9.83, SD = 3.50), no significant difference was found. Additionally, the mean score for medication adherence was 7.59 (SD = 1.34), ranging from 0 to 8. For the follow-up sample, 90.78% of them were classified as low-level SMBG, which means that they tested blood glucose less than twice a week. There were no significant differences between the respondents with HbA1c levels < 7% and respondents with HbA1c levels ≥ 7% in terms of medication adherence, DSM in diet, and SMBG. As reported in Table 4 and Supplementary Table S2, medication adherence and DSM factors did not influence HbA1c levels significantly in either the multiple linear regression model or the logistic regression model.

**Table 3:** Relationships between HbA1c levels and clinical outcomes among 206 people with T2DM at T2.

	HbA_1_c level < 7% (n = 119)	HbA_1_c level ≥ 7% (n = 87)	*p*^*a*^
Diabetes knowledge at baseline	10.25 ± 3.36	9.83 ± 3.50	0.380
Medication adherence			
Adherence	17 (15.74)	13 (15.29)	0.932
Non-adherence	91 (84.26)	72 (84.71)	
Diabetes self-management (exercise)			
Low level	55 (46.22)	27 (31.03)	0.028
High level	64 (53.78)	60 (68.97)	
Diabetes self-management (diet)	25.72 ± 6.72	26.62 ± 6.60	0.345
Diabetes self-management (SMBG)*			
Low level	110 (92.44)	77 (88.51)	0.335
High level	9 (7.56)	10 (11.49)	

Data are presented as mean ± SD or number (percent). * Diabetes self-management was evaluated from three dimensions: exercise, diet, and the self-monitoring of blood glucose (SMBG). ^a^ Based on a Student's t-test or Pearson’s Chi-squared test.

**Table 4:** Hierarchical regression analyses predicting HbA1c levels at T2.

Variable	Model 1	Model 2	Model 3	Model 4	Model 5
	Coefficients (95% CI)	Coefficients (95% CI)	Coefficients (95% CI)	Coefficients (95% CI)	Coefficients (95% CI)
Sex					
Male (ref)					
Female	−0.878***	−0.645***	−0.675***	−0.675***	−0.669***
	(−1.374 to −0.382)	(−0.971 to −0.319)	(−0.994 to −0.356)	(−0.994 to −0.355)	(−0.989 to −0.348)
Age	−0.033*	−0.023*	−0.023*	−0.023*	−0.024*
	(−0.064 to −0.003)	(−0.043 to −0.003)	(−0.043 to −0.004)	(−0.043 to −0.004)	(−0.044 to −0.005)
Employment status					
Other Status (ref)					
Farming	−0.440	−0.309*	−0.330*	−0.338*	−0.296*
	(−0.893 to 0.013)	(−0.608 to −0.010)	(−0.622 to −0.037)	(−0.632 to −0.044)	(−0.592 to −0.000)
Annual household income (yuan)^a^					
≤ 5,000 (ref)					
5,000–10,000	0.338	0.386	0.457	0.462	0.473*
	(−0.407 to 1.084)	(−0.090 to 0.862)	(−0.010 to 0.925)	(−0.006 to 0.930)	(0.006 to 0.940)
10,000–20,000	−0.000	0.076	0.146	0.147	0.188
	(−0.725 to 0.725)	(−0.392 to 0.544)	(−0.313 to 0.606)	(−0.313 to 0.608)	(−0.273 to 0.649)
20,000–50,000	−0.147	0.080	0.150	0.153	0.183
	(−0.879 to 0.585)	(−0.393 to 0.552)	(−0.314 to 0.614)	(−0.312 to 0.618)	(−0.283 to 0.650)
> 50,000	−0.484	−0.243	−0.153	−0.158	−0.110
	(−1.283 to 0.315)	(−0.760 to 0.274)	(−0.661 to 0.356)	(−0.668 to 0.351)	(−0.622 to 0.402)
HbA1c at baseline		0.832***	0.842***	0.841***	0.828***
		(0.729 to 0.935)	(0.741 to 0.943)	(0.740 to 0.942)	(0.726 to 0.930)
Diabetes knowledge at baseline			−0.063**	−0.065**	−0.062**
			(−0.105 to −0.021)	(−0.106 to −0.023)	(−0.104 to −0.020)
Medication adherence					
Adherence (ref)					
Non-adherence				−0.138	−0.167
				(−0.508 to 0.232)	(−0.537 to 0.202)
Diabetes self-management (exercise)					
Low level (ref)					
High level					0.213
					(−0.074 to 0.499)
Diabetes self-management (diet)					
Low level (ref)					
High level					0.005
					(−0.017 to 0.027)
Diabetes self-management (SMBG)					
Low level (ref)					
High level					0.278
					(−0.234 to 0.790)
Constant	10.138***	2.562*	3.107**	3.284**	3.078**
	(7.632 to 12.645)	(0.441 to 4.682)	(1.003 to 5.211)	(1.124 to 5.443)	(0.820 to 5.337)
R-squared	0.105	0.654	0.671	0.672	0.681
Adjust R-squared	0.054	0.620	0.637	0.636	0.638
N	206	206	206	206	206

ci in parentheses.

***p < 0.001; **p < 0.01; *p < 0.05.

a: The exchange rate of Chinese Yuan in US Dollars was 0.15715 USD for 1 CNY. Model 1 was also adjusted for education level, marital status. Models 2, 3, 4, and 5 were also adjusted for demographic characteristics (education level, marital status), and clinical characteristics (duration of T2DM, number of complications, family history, body mass index, and hypoglycemia). The full version of Table 4 was shown in the appendix.

### Diabetes knowledge as a predictor of HbA1c levels at T2

As shown in Table 4, in Model 1, the inclusion of sex, age, education, marital status, employment status, and annual household income account for 10.5% of the total variance of HbA1c levels (T2). The combined effect of clinical outcomes at baseline explained an additional 54.9% of the total variance in Model 2. Model 3 indicated that diabetes knowledge was a crucial predictor of HbA1c levels (β = −0.063, *p* < 0.01), by itself, explaining an additional 1.7% of the total variance. In Model 4 and Model 5, medication adherence and DSM were entered into the multiple regression analyses, and diabetes knowledge was still a significant predictor factor for HbA1c levels (β = −0.065, *p* < 0.01; β = −0.062, *p* < 0.01). Besides, sex (β = −0.669, *p* < 0.001), age (β = −0.024, *p* < 0.05), employment status (β = −0.296, *p* < 0.05), annual household income (β = 0.473, *p* < 0.05), and HbA1c levels (T1) (β = 0.828, *p* < 0.001) significantly influenced the HbA1c levels at T2.

To further investigate the stability of the relationship between diabetes knowledge and HbA1c levels and to explore which factors influenced successful glycemic control, we divided the participants into two groups according to their HbA1c levels (T2), with a cut-off point of HbA1c levels less than 7%^46^. If the HbA1c level(T2) was less than 7%, he or she had successful glycemic control, and not vice versa. Similarly, the covariates were input into the logistic regression model in five steps. Supplementary Table S1 showed that sex, marital status, employment status, HbA1c levels(T1), and diabetes knowledge significantly influenced HbA1c levels(T2). Female respondents were more likely to control glycemia successfully. The married respondents had higher risks of unsuccessful glycemic control than single respondents. Compared with people engaged in other jobs, respondents who were farmers were more likely to control glycemia successfully. Apparently, in the multiple logistic regression model, those respondents who had a higher score of diabetes knowledge would have a greater chance of controlling glycemia successfully.

## Discussion

In this study, we investigated the correlation between diabetes knowledge and HbA1c levels in people with T2DM based on tracking data in rural China. We found that diabetes knowledge could predict HbA1c levels before and after adjusting for sociodemographic, clinical, and behavioral variables. Furthermore, multiple logistic regression analysis confirmed the results. It was consistent with the results of a study also conducted in Jiangsu Province, which found that improving diabetes knowledge helps lower FBG levels after a one-year educational intervention^47^.

The results of this study may suggest that improving diabetes knowledge leads to a decrease in HbA1c levels. The correlation can be explained by knowledge, attitude, and practices (KAP), which has been applied to health education practice since the 1960s. For people with T2DM, receiving ongoing diabetes health education can improve their understanding of diabetes and help them establish an active attitude toward treatments. Patients’ active attitudes may enable them to change their DSM behaviors and further influence the HbA1c levels.

However, the coefficient of diabetes knowledge may not be very high in the multiple linear regression model. Reviewing previous studies, a study estimated that every time the patients answered one more question, the HbA1c level decreased by 0.239^48^. Another study found that 82% of participants had HbA1c > 7%, which was associated with poor diabetes knowledge. Additionally, a systematic review concluded that continuous and regular education could result in a mean reduction of 2.02% for HbA1c among Chinese patients^49^. In contrast, a study conducted in an urban area in China found that there was no significant difference in the knowledge scores between people with HbA1c < 7% and those with HbA1c ≥ 7%^32^. Another study revealed that after a two-year educational intervention, there was no significant difference in FBG levels between treatment and control groups^50^. These findings were inconsistent, which suggested that the relationship between diabetes knowledge and glycemic control is worthy of further study.

There are two reasons why the coefficient of diabetes knowledge is not very high. On the one hand, as in previous studies^51^, there is still a large gap between knowledge and behaviors related to glycemic control, and attitudes or undiscovered factors may play an essential role in the process. On the other hand, several sociodemographic variables also influenced the HbA1c level significantly in Model 5. First, female patients’ HbA1c levels (T2) were lower than those of males, and female patients were more likely to successfully control glycemia. It was not consistent with results obtained from other studies^52–55^. Some studies found that there was no sex difference in glycemic control^54,55^, and other studies concluded that females were less likely to achieve the target HbA1c of < 7%^52,53^. Although females had better self-care and high levels of adherence^53^, depression was more common in females than males, which made it more difficult for women to successfully control glycemia^56^. These studies were mostly conducted in urban areas; however, the setting of this study was a rural area. There are many differences between urban and rural areas, such as economic and cultural factors, which may influence the sex differences in glycemic control. In rural China, females need to do both farm work and housework, while males are mainly busy with farm work and rarely do housework. Thus, the total amount of exercise of females may be higher than that of males, which increases the possibility of females achieving success in glycemic control. Second, older patients may have lower HbA1c levels since older patients have more time to focus on their health, while younger patients are busy dealing with work and family. Third, patients who are farming had lower HbA1c levels than patients in other jobs because farmers perform more physical activities than other jobs. In addition, compared with people whose monthly income is less than 5,000 yuan, people whose monthly income is 5,000–10,000 yuan had lower HbA1c levels, as patients were capable of paying medical bills.

Similarly, sex, employment status, and diabetes knowledge significantly influenced HbA1c levels in the logistic regression model. Furthermore, married respondents had higher risks of unsuccessful glycemic control than single respondents. It seems probable that when a couple has a conflict, the DSM behavior of one of them will worsen^57^. Therefore, it seemed that single patients were more likely to achieve a target HbA1c of < 7%.

Notably, as reported in Table 1, the people with T2DM had poor diabetes knowledge in rural China. This was because they had a low education level, and some of them were even illiterate. In addition, the patients’ mean age was older than 60 years old, which was related to poor diabetes knowledge, as reported by a qualitative study^58^. As recent research reported, patients’ low level of diabetes knowledge is an objective phenomenon in rural China^59^. Our finding is in line with a study in Thailand^60^, which found that people with T2DM also had poor diabetes knowledge. Based on the facts of poor diabetes knowledge, improving diabetes knowledge levels may enable patients to realize the severity of T2DM and promote behavior change.

In addition, we found that medication adherence and DSM factors did not influence HbA1c levels significantly in either the multiple linear regression or logistic regression model. As reported in Table 3, most participants had poor medication adherence and a low level of DSM due to a lack of diabetes knowledge. It is noted that SMBG was not associated with HbA1c levels (Supplementary Table S4) in this study, which was inconsistent with the meta-analysis research^61^. Actually, the relationship between SMBG and HbA1c levels remains unclear. A recent randomized trial found there were no clinically or statistically significant differences at 1 year in glycemic control between patients who performed SMBG compared with those who did not perform SMBG^62^. Theoretically, what is important is the patient’s behavior change based on the results of SMBG that could influence glycemic control, rather than SMBG itself. Therefore, the relationship between these two variables and the potential mechanism should be explored in the future.

There are several limitations to the current study. First, only 64.17% of participants returned for the second measurement of HbA1c. Although more men declined the second visit (Table 1), no significant difference was found between follow-up men and loss to follow-up men in terms of diabetes knowledge, and neither did women (Supplementary Table S3). Therefore, the follow-up samples were unbiased and loss to follow-up did not affect the stability of the relationship between diabetes knowledge and HbA1c levels. Second, the current study relies on a self-report measure of medication adherence and DSM, social desirability bias and recall bias may still exist. More objective measurements of DSM should be used in the future. Third, only one district was selected in this study, future work will be extended to other sites so that the universality and differences in the relationship between diabetes knowledge and HbA1c levels can be tested.

Concurrently, this study has significant strengths. First, tracking data were used to explore the correlation between the independent variable and dependent variable, which made causality more plausible. Second, HbA1c was used to assess patients’ average blood glucose concentrations during the preceding 2–3 months, which were more stable than others. Third, people with T2DM in rural areas were chosen as the study population, who were in urgent need of improving DSM behaviors but were rarely concerned, which has practical significance.

## Conclusions

This study provided longitudinal evidence for the effects of diabetes knowledge on HbA1c levels in patients with low education levels, which indicated that interventions focusing on diabetes knowledge need to be strengthened in rural China.

The acquisition of knowledge has been played down for several decades in community chronic disease management. The findings presented important evidence, that knowledge acquisition may have an important role, which may have some implications for the policy of chronic disease management for low- and middle-income countries. Improving diabetes knowledge need to be strengthened for patients with low education level in rural China, which help improve outcomes and reduce the disease burden.

### Supplementary Information

Supplementary Information.

## Data Availability

The datasets used and/or analysed during the current study are available from the corresponding author on reasonable request.

## Abbreviations

T2DM: Type 2 diabetes mellitus
HbA1c: Glycated haemoglobin
BMI: Body mass index
IDF: International Diabetes Federation
FBG: Fasting blood glucose
DKN: Original version of the diabetes knowledge scales
SDSCA: Original version of the diabetes self-care activities measure
DSM: Diabetes self-management
