# Supplementary material for: Diabetes knowledge predicts HbA1c levels of people with type 2 diabetes mellitus in rural China: a ten-month follow-up study
Source: Sci Rep. 2023 Oct 25;13:18248. doi: 10.1038/s41598-023-45312-y (PMC10600128; doi:10.1038/s41598-023-45312-y)
Supplement: Supplementary file 1 — Supplementary Information. [file 41598_2023_45312_MOESM1_ESM.docx]

**Diabetes Knowledge Predicts HbA1c Levels of People with Type 2 Diabetes Mellitus in Rural China: A Ten-Month Follow-Up Study**

Xiaoying Wang^a,1^, Bo Tian ^a,1^, Shengfa Zhang^b^, Jinsui Zhang^c^, Weiwei Wang^d^, Jina Li^e^, Weiping Yang^f^,Yuchen Wang^g^, Weijun Zhang^a*^

**ORCID iDs**

Xiaoying Wang 0000-0003-0670-9988

Weijun Zhang 0000-0003-3436-3371

**Address for each author**

^a^ School of Social Development and Public Policy, Center for Behavior Health, Beijing Normal University, Beijing, China.

^b^ National Population Heath Data Center, Chinese Academy of Medical Sciences and Peking Union Medical College, Beijing, China.

^c^ School of Public Health, Fudan University, Shanghai, China

^d^ School of Sociology and Population Studies, Renmin University of China, Beijing, China

^e^ State Key Laboratory of Cognitive Neuroscience and Learning, Beijing Normal University, Beijing,

China.

^f^ Health Commission of Dafeng District, Jiangsu Province, China.

^g^ North China Electric Power University, Beijing, China

^1.^ These authors contributed equally to this work.

^^[[1]](#footnote-1)^*^ Corresponding author at 19, Xinjiekou Wai Street, Beijing 100875, China.

Tel.: +86 10 58805031; fax: +86 10 58800366.

E-mail address: [zwj@bnu.edu.cn](mailto:zwj@bnu.edu.cn)

**SUPPLEMENTARY MATERIAL**

**The process of modifying the DKN scale**

First, the original version of the DKN scale was translated into Chinese by a professor and two postgraduates. Second, several Chinese scientists in the field of chronic diseases reviewed and revised the Chinese version of DKN scale. Third, the revised Chinese version of the instrument was blindly translated back into English by two postgraduates at the School of Foreign Languages and Literatures. The two English versions of the instruments (the original and the back-translation) were reviewed and compared by a professor at the School of Foreign Languages and Literatures. No discrepancies were identified.

To test the revised version of the instrument, a pilot study was carried out and 69 people with T2DM completed the scale in 6 villages from DaFeng District, Jiangsu province. Based on the pilot study, some changes were made in the final version as follows: (1) the order of items was adjusted to improve the response rate; (2) the options for the last two items were changed to food that Chinese people are more likely to eat, for example, the word “bread” was changed to “steamed bun”; (3) the units of measurement in the options were replaced given participants’ low education level and cultural diversity, for instance, “3/4 cup” was replaced by “1 cup” and “5 oz” was replaced by “1 cup” respectively.

**Supplementary Table S1: the full version of Table 4**

**Hierarchical regression analyses predicting HbA1c levels at T2**

|  | **Model 1** | **Model 2** | **Model 3** | **Model 4** | **Model 5** |
| --- | --- | --- | --- | --- | --- |
| **Variable** | **Coefficients**  **(95% CI)** | **Coefficients**  **(95% CI)** | **Coefficients**  **(95% CI)** | **Coefficients**  **(95% CI)** | **Coefficients**  **(95% CI)** |
| **Sex** |  |  |  |  |  |
| Male (ref) |  |  |  |  |  |
| Female | -0.878*** | -0.645*** | -0.675*** | -0.675*** | -0.669*** |
|  | (-1.374 – -0.382) | (-0.971 – -0.319) | (-0.994 – -0.356) | (-0.994 – -0.355) | (-0.989 – -0.348) |
| **Age** | -0.033* | -0.023* | -0.023* | -0.023* | -0.024* |
|  | (-0.064 – -0.003) | (-0.043 – -0.003) | (-0.043 – -0.004) | (-0.043 – -0.004) | (-0.044 – -0.005) |
| **Education Level** |  |  |  |  |  |
| Uneducated (ref) |  |  |  |  |  |
| Primary school | 0.065 | 0.163 | 0.232 | 0.246 | 0.251 |
|  | (-0.519 – 0.649) | (-0.215 – 0.541) | (-0.141 – 0.604) | (-0.129 – 0.621) | (-0.123 – 0.625) |
| Junior high school and above | -0.495 | -0.269 | -0.124 | -0.121 | -0.156 |
|  | (-1.117 – 0.127) | (-0.670 – 0.133) | (-0.528 – 0.280) | (-0.526 – 0.283) | (-0.563 – 0.252) |
| **Marital Status** |  |  |  |  |  |
| Single (ref) |  |  |  |  |  |
| Married | 0.160 | 0.387 | 0.379 | 0.380 | 0.407 |
|  | (-0.542 – 0.861) | (-0.063 – 0.838) | (-0.061 – 0.820) | (-0.061 – 0.821) | (-0.034 – 0.848) |
| **Employment Status** |  |  |  |  |  |
| Other Status (ref) |  |  |  |  |  |
| Farming | -0.440 | -0.309* | -0.330* | -0.338* | -0.296* |
|  | (-0.893 – 0.013) | (-0.608 – -0.010) | (-0.622 – -0.037) | (-0.632 – -0.044) | (-0.592 – -0.000) |
| **Income over the past year (yuan)^a^** |  |  |  |  |  |
| ≤5,000 (ref) |  |  |  |  |  |
| 5,000~10,000 | 0.338 | 0.386 | 0.457 | 0.462 | 0.473* |
|  | (-0.407 – 1.084) | (-0.090 – 0.862) | (-0.010 – 0.925) | (-0.006 – 0.930) | (0.006 – 0.940) |
| 10,000~20,000 | -0.000 | 0.076 | 0.146 | 0.147 | 0.188 |
|  | (-0.725 – 0.725) | (-0.392 – 0.544) | (-0.313 – 0.606) | (-0.313 – 0.608) | (-0.273 – 0.649) |
| 20,000~50,000 | -0.147 | 0.080 | 0.150 | 0.153 | 0.183 |
|  | (-0.879 – 0.585) | (-0.393 – 0.552) | (-0.314 – 0.614) | (-0.312 – 0.618) | (-0.283 – 0.650) |
| >50,000 | -0.484 | -0.243 | -0.153 | -0.158 | -0.110 |
|  | (-1.283 – 0.315) | (-0.760 – 0.274) | (-0.661 – 0.356) | (-0.668 – 0.351) | (-0.622 – 0.402) |
| **Duration** |  | 0.001 | 0.001 | 0.001 | 0.000 |
|  |  | (-0.025 – 0.028) | (-0.025 – 0.027) | (-0.025 – 0.027) | (-0.026 – 0.027) |
| **The number of complications** | |  |  |  |  |
| 0 (ref) |  |  |  |  |  |
| 1 |  | -0.197 | -0.182 | -0.180 | -0.179 |
|  |  | (-0.529 – 0.135) | (-0.506 – 0.143) | (-0.505 – 0.145) | (-0.508 – 0.150) |
| ≥2 |  | 0.166 | 0.212 | 0.214 | 0.248 |
|  |  | (-0.272 – 0.605) | (-0.218 – 0.642) | (-0.217 – 0.644) | (-0.183 – 0.678) |
| **Family history of diabetes** |  |  |  |  |  |
| Yes (ref) |  |  |  |  |  |
| No |  | 0.159 | 0.169 | 0.178 | 0.171 |
|  |  | (-0.179 – 0.498) | (-0.162 – 0.500) | (-0.154 – 0.510) | (-0.164 – 0.506) |
| **BMI** |  | -0.013 | -0.015 | -0.017 | -0.016 |
|  |  | (-0.052 – 0.027) | (-0.054 – 0.024) | (-0.056 – 0.022) | (-0.055 – 0.023) |
| **Hypoglycemia** |  |  |  |  |  |
| Yes (ref) |  |  |  |  |  |
| No |  | 0.088 | 0.067 | 0.069 | 0.066 |
|  |  | (-0.207 – 0.382) | (-0.221 – 0.355) | (-0.220 – 0.357) | (-0.221 – 0.354) |
| **HbA1c at baseline** |  | 0.832*** | 0.842*** | 0.841*** | 0.828*** |
|  |  | (0.729 – 0.935) | (0.741 – 0.943) | (0.740 – 0.942) | (0.726 – 0.930) |
| **Diabetes knowledge at baseline** |  |  | -0.063** | -0.065** | -0.062** |
|  |  |  | (-0.105 – -0.021) | (-0.106 – -0.023) | (-0.104 – -0.020) |
| **Medication adherence** |  |  |  |  |  |
| Adherence (ref) |  |  |  |  |  |
| Non-adherence |  |  |  | -0.138 | -0.167 |
|  |  |  |  | (-0.508 – 0.232) | (-0.537 – 0.202) |
| **Diabetes self-management (exercise)** | |  |  |  |  |
| Low level (ref) |  |  |  |  |  |
| High level |  |  |  |  | 0.213 |
|  |  |  |  |  | (-0.074 – 0.499) |
| **Diabetes self-management (diet)** | |  |  |  |  |
| Low level (ref) |  |  |  |  |  |
| High level |  |  |  |  | 0.005 |
|  |  |  |  |  | (-0.017 – 0.027) |
| **Diabetes self-management (SMBG)** | |  |  |  |  |
| Low level (ref) |  |  |  |  |  |
| High level |  |  |  |  | 0.278 |
|  |  |  |  |  | (-0.234 – 0.790) |
| Constant | 10.138*** | 2.562* | 3.107** | 3.284** | 3.078** |
|  | (7.632 – 12.645) | (0.441 – 4.682) | (1.003 – 5.211) | (1.124 – 5.443) | (0.820 – 5.337) |
| R-squared | 0.105 | 0.654 | 0.671 | 0.672 | 0.681 |
| Adjust R-squared | 0.054 | 0.620 | 0.637 | 0.636 | 0.638 |
| N | 206 | 206 | 206 | 206 | 206 |

Note: ci in parentheses

*** p<0.001, ** p<0.01, * p<0.05

a: The exchange rate of Chinese Yuan in US Dollars was 0.15715 USD for 1 CNY

**Supplementary Table S2: Logistic analyses predicting HbA1c levels at T2**

|  | Model 1 | Model 2 | Model 3 | Model 4 | Model 5 |
| --- | --- | --- | --- | --- | --- |
| VARIABLES | OR | OR | OR | OR | OR |
| **Gender** |  |  |  |  |  |
| Male |  |  |  |  |  |
| Female | 2.045* | 2.933* | 3.390* | 3.244* | 3.462* |
|  | (1.040 – 4.022) | (1.050 – 8.192) | (1.164 – 9.876) | (1.107 – 9.505) | (1.166 – 10.280) |
| **Age** | 1.029 | 1.052 | 1.054 | 1.052 | 1.054 |
|  | (0.988 – 1.072) | (0.993 – 1.114) | (0.992 – 1.119) | (0.988 – 1.121) | (0.989 – 1.123) |
| **Education Level** |  |  |  |  |  |
| Uneducated |  |  |  |  |  |
| Primary school | 0.872 | 0.592 | 0.445 | 0.496 | 0.458 |
|  | (0.397 – 1.913) | (0.197 – 1.777) | (0.142 – 1.395) | (0.155 – 1.593) | (0.137 – 1.525) |
| Junior high school and above | 1.160 | 1.287 | 0.753 | 0.658 | 0.695 |
|  | (0.493 – 2.732) | (0.381 – 4.346) | (0.203 – 2.795) | (0.180 – 2.411) | (0.185 – 2.615) |
| **Marital Status** |  |  |  |  |  |
| Single |  |  |  |  |  |
| Married | 0.666 | 0.244* | 0.229* | 0.241* | 0.202* |
|  | (0.251 – 1.765) | (0.064 – 0.926) | (0.059 – 0.897) | (0.062 – 0.938) | (0.050 – 0.821) |
| **Employment Status** |  |  |  |  |  |
| Other Status |  |  |  |  |  |
| Farming | 1.982* | 2.970* | 3.612** | 3.221* | 2.995* |
|  | (1.067 – 3.682) | (1.179 – 7.481) | (1.387 – 9.405) | (1.233 – 8.417) | (1.144 – 7.839) |
| **Income over the past year (yuan)^a^** |  |  |  |  |  |
| ≤5,000 |  |  |  |  |  |
| 5,000~10,000 | 0.859 | 0.789 | 0.697 | 0.642 | 0.638 |
|  | (0.315 – 2.342) | (0.197 – 3.158) | (0.163 – 2.976) | (0.148 – 2.795) | (0.140 – 2.913) |
| 10,000~20,000 | 0.865 | 1.085 | 0.866 | 0.765 | 0.758 |
|  | (0.333 – 2.245) | (0.280 – 4.199) | (0.209 – 3.579) | (0.183 – 3.190) | (0.181 – 3.175) |
| 20,000~50,000 | 1.505 | 2.518 | 1.897 | 1.689 | 1.562 |
|  | (0.564 – 4.019) | (0.608 – 10.431) | (0.430 – 8.379) | (0.373 – 7.643) | (0.340 – 7.171) |
| >50,000 | 1.639 | 2.682 | 2.001 | 1.720 | 1.609 |
|  | (0.564 – 4.767) | (0.585 – 12.308) | (0.416 – 9.625) | (0.352 – 8.397) | (0.321 – 8.060) |
| **Duration** |  | 1.017 | 1.025 | 1.027 | 1.027 |
|  |  | (0.947 – 1.092) | (0.953 – 1.102) | (0.954 – 1.106) | (0.952 – 1.108) |
| **The number of complications** |  |  |  |  |  |
| 0 |  |  |  |  |  |
| 1 |  | 1.110 | 1.057 | 0.887 | 0.927 |
|  |  | (0.421 – 2.926) | (0.389 – 2.873) | (0.316 – 2.493) | (0.318 – 2.704) |
| ≥2 |  | 0.885 | 0.779 | 0.693 | 0.651 |
|  |  | (0.226 – 3.462) | (0.189 – 3.216) | (0.167 – 2.875) | (0.150 – 2.824) |
| **Family history** |  |  |  |  |  |
| Yes |  |  |  |  |  |
| No |  | 0.654 | 0.675 | 0.663 | 0.657 |
|  |  | (0.247 – 1.729) | (0.251 – 1.813) | (0.243 – 1.813) | (0.234 – 1.848) |
| **BMI** |  | 1.012 | 1.036 | 1.061 | 1.056 |
|  |  | (0.901 – 1.137) | (0.918 – 1.170) | (0.936 – 1.203) | (0.929 – 1.201) |
| **Hypoglycemia** |  |  |  |  |  |
| Yes |  |  |  |  |  |
| No |  | 1.083 | 1.292 | 1.277 | 1.309 |
|  |  | (0.447 – 2.627) | (0.514 – 3.247) | (0.501 – 3.251) | (0.500 – 3.427) |
| **HbA1c at baseline** |  | 0.153*** | 0.140*** | 0.149*** | 0.143*** |
|  |  | (0.090 – 0.260) | (0.081 – 0.242) | (0.086 – 0.258) | (0.080 – 0.254) |
| **Diabetes knowledge at baseline** |  |  | 1.197* | 1.191* | 1.202* |
|  |  |  | (1.040 – 1.376) | (1.034 – 1.371) | (1.036 – 1.394) |
| **Medication adherence** |  |  |  |  |  |
| Adherence |  |  |  |  |  |
| Non-adherence |  |  |  | 1.063 | 1.063 |
|  |  |  |  | (0.266 – 4.246) | (0.266 – 4.247) |
| **Diabetes self-management (exercise)** |  |  |  |  |  |
| Low level |  |  |  |  |  |
| High level |  |  |  |  | 0.688 |
|  |  |  |  |  | (0.281 – 1.684) |
| **Diabetes self-management (diet)** |  |  |  |  |  |
| Low level |  |  |  |  |  |
| High level |  |  |  |  | 0.985 |
|  |  |  |  |  | (0.916 – 1.059) |
| **Diabetes self-management (SMBG)** |  |  |  |  |  |
| Low level |  |  |  |  |  |
| High level |  |  |  |  | 0.333 |
|  |  |  |  |  | (0.073 – 1.511) |
| Pseudo R-squared | 0.046 | 0.436 | 0.461 | 0.453 | 0.466 |

Note: ci in parentheses

*** p<0.001, ** p<0.01, * p<0.05

a: The exchange rate of Chinese Yuan in US Dollars was 0.15715 USD for 1 CNY.

**Supplementary Table S3: The comparison of patient’s diabetes knowledge between the follow-up samples and lost to follow-up samples**

|  | **Follow-up samples (n=206)** | **Lost to follow-up samples (n=115)** | **p value** |
| --- | --- | --- | --- |
| **Gender** |  |  |  |
| Male | 10.74±0.43 | 10.62±0.55 | t=-0.184, p=0.854 |
| Female | 9.73±0.28 | 9.02±0.53 | t=-1.273, p=0.205 |

Data are presented as mean ± SD or number (percent).

**Supplementary Table S4: The comparison of HbA1c levels at T2 between two levels of SMBG**

|  | **HbA1c levels at T2**  **(n=206)** | **p value** |
| --- | --- | --- |
| **SMBG** |  |  |
| Low level | 7.04±0.12 | t=-0.409, p=0.683 |
| High level | 7.19±0.34 |  |

Data are presented as mean ± SD or number (percent).

1. [↑](#footnote-ref-1)
